# Controlling the signal: Practical privacy protection of genomic data sharing through Beacon services

**DOI:** 10.1186/s12920-017-0282-1

**Published:** 2017-07-26

**Authors:** Zhiyu Wan, Yevgeniy Vorobeychik, Murat Kantarcioglu, Bradley Malin

**Affiliations:** 1 Department of Electrical Engineering and Computer Science, Vanderbilt University, 2525 West End Avenue, Suite 800, 37203 Nashville, TN USA; 2 Department of Biomedical Informatics, Vanderbilt University, Nashville, TN USA; 3 Department of Computer Science, University of Texas at Dallas, Richardson, TX USA; 4 Department of Biostatistics, Vanderbilt University, Nashville, TN USA

**Keywords:** Genomic databases, Beacon service, Data sharing, Privacy, Perturbation, iDASH challenge

## Abstract

**Background:**

Genomic data is increasingly collected by a wide array of organizations. As such, there is a growing demand to make summary information about such collections available more widely. However, over the past decade, a series of investigations have shown that attacks, rooted in statistical inference methods, can be applied to discern the presence of a known individual’s DNA sequence in the pool of subjects. Recently, it was shown that the Beacon Project of the Global Alliance for Genomics and Health, a web service for querying about the presence (or absence) of a specific allele, was vulnerable. The Integrating Data for Analysis, Anonymization, and Sharing (iDASH) Center modeled a track in their third Privacy Protection Challenge on how to mitigate the Beacon vulnerability. We developed the winning solution for this track.

**Methods:**

This paper describes our computational method to optimize the tradeoff between the utility and the privacy of the Beacon service. We generalize the genomic data sharing problem beyond that which was introduced in the iDASH Challenge to be more representative of real world scenarios to allow for a more comprehensive evaluation. We then conduct a sensitivity analysis of our method with respect to several state-of-the-art methods using a dataset of 400,000 positions in Chromosome 10 for 500 individuals from Phase 3 of the 1000 Genomes Project. All methods are evaluated for utility, privacy and efficiency.

**Results:**

Our method achieves better performance than all state-of-the-art methods, irrespective of how key factors (e.g., the allele frequency in the population, the size of the pool and utility weights) change from the original parameters of the problem. We further illustrate that it is possible for our method to exhibit subpar performance under special cases of allele query sequences. However, we show our method can be extended to address this issue when the query sequence is fixed and known a priori to the data custodian, so that they may plan stage their responses accordingly.

**Conclusions:**

This research shows that it is possible to thwart the attack on Beacon services, without substantially altering the utility of the system, using computational methods. The method we initially developed is limited by the design of the scenario and evaluation protocol for the iDASH Challenge; however, it can be improved by allowing the data custodian to act in a staged manner.

## Background

Genomic data is increasingly collected by a wide array of organizations [1], ranging from direct-to-consumer genomics companies [2] to clinical institutions [3, 4]. This data serves as the basis of discovery-driven research [5, 6] and, more recently, for personalized medicine programs [7, 8]. However, as the quantity and coverage of genomic data grow, so too does the chance for the discovery and reporting of rare alleles [9, 10]. This is challenging for researchers and clinicians who aim to discern if such an allele (or combination of alleles across the genome) is meaningful with respect to an individual’s phenotypic status or should influence the design of a personalized treatment regimen. To mitigate uncertainty, there is a desire to open data held by one organization to those who may need it elsewhere [11, 12]. While there are some initiatives, like the Personal Genome Project [13], that freely and publicly share genomic data linked to phenomic data, the existence of such systems does not necessarily translate into a large number of participants [14]. There are numerous reasons why individuals may not contribute their data to such programs, one of which is a privacy concern that the data would be misused or abused in some way [15, 16]. To mitigate privacy risks, data custodians have turned towards sharing summary level data about the pool of individuals who were in a study or were treated in a clinical setting.

The practice of summary data sharing began on a large scale in the mid-2000’s, with programs like the Database of Genotypes and Phenotypes (dbGaP) at the National Institutes of Health [17], which aimed to standardize and centralize genomic data, making it easier to access. Summary statistics about the allele rates were made publicly accessible over the Internet because it was assumed that the privacy risks for such data were minimal. Yet in 2008, Homer and colleagues [18] demonstrated that an adversary could apply a statistical inference attack to discern the presence of a known individual’s DNA sequence in the pool of subjects. This was specifically accomplished by measuring the distance between an individual’s sequence to the allele rates exhibited by the pool versus some reference population, such as the International Haplotype Mapping Program [19] or 1000 Genomes [20]. When the target was deemed to be sufficiently biased towards the pool, the adversary could assign the hallmarks of the pool, such as membership in a specific group for case-control study (e.g., individuals positively-diagnosed with a sexually transmitted disease). As an artifact of this demonstration, the NIH, Wellcome Trust, and other genomic data custodians restricted access to summary-level genomic data [21, 22]. Since the initial attack, there have been a number of advancements in pool detection methodology (e.g., [23–27]).

As such inference methods evolved, the Global Alliance for Genomics and Health (GA4GH) formed to facilitate the sharing of genomic and health data in a federated manner [28]. In light of the known attacks, GA4GH created the Beacon Project, which enables data custodians to respond to queries through a web service (i.e., a beacon) about the presence/absence of a specific allele [29]. For instance, the Beacon service could respond yes/no to a question like, “Do you have any genomes with nucleotide A at position 121,212,028 on chromosome 10?” When the answer was affirmative, the requesting system user would learn that the variant in question may not be unique (i.e., because it was observed in a genome collected elsewhere) and that it might be worth pursuing further investigation into its meaning (possibly with the assistance of the answering data custodian).

Though it obscures allele rates, in late 2015, the Beacon service was also shown to be vulnerable to a statistical inference attack. Specifically, Shringarpure and Bustamante (SB) described the statistical theory behind the attack and illustrated how it might require no more than 5,000 responses to infer an individual’s, or their relatives’, membership in the pool [30].

Given the increasing adoption of Beacon, the Integrating Data for Analysis, Anonymization, and Sharing (iDASH) National Center for Biomedical Computing allocated one of the three tracks of their 2016 Genomic Privacy Protection Challenge to explicitly focus on this vulnerability. The organizers formulated the problem as, “Given a sample Beacon database, we will ask [the] participating team to develop solutions to mitigate the Bustamante attack. We will evaluate each algorithm based on the maximum number of correct queries that it can respond [to] before any individual can be re-identified by the Bustamante attack.” [31] A subset of the authors of this paper developed the winning solution to this challenge. While this paper provides the details behind this solution, we have further extended our initial analysis to illustrate its limits as well as introduce alternative formulations of the problem to evolve the investigation into a setting closer to the real world.

The specific contributions of this paper include:

1. We introduce a method that simultaneously optimizes the privacy (based on the SB attack as augmented by the iDASH Challenge organizers) and the utility of the system;
2. We generalize the genomic data sharing problem to be more representative of scenarios in which beacons will actually be deployed; and
3. We provide a sensitivity and robustness analysis of our method under various parameterizations of the variables relied upon by the iDASH Challenge.

## Methods

### The iDASH challenge

The goal of the first track of the 2016 iDASH Challenge was to mitigate an augmented version of the SB attack. The problem was how to find such a strategy for the genomic data custodian. This section begins with a description of the attack model and then models the data custodian’s strategy as an optimization problem. In this setting, the attacker is defined as a malicious user launching the SB attack. The defender, by contrast, is defined as the data custodian sharing the genomic data while mitigating the SB attack.

### iDASH variation of the SB attack

Given the binary genomic summary statistics of a pool of genomes (i.e., the beacon), the attacker relies upon a likelihood ratio test (LRT) to infer whether a targeted genome is in the pool or not. The null hypothesis, *H*_0_, is that the targeted genome is not in the beacon, While the alternative hypothesis, *H*_1_, is that the targeted genome is in the beacon. The attack model used in the iDASH Challenge is based on the SB attack, but it is amended to allow the attacker to know the alternative allele frequency (AAF) of all single nucleotide variants (SNVs) in the underlying population of the beacon. Here, AAF is the frequency at which the alternative allele occurs in a given population. The alternative allele is defined as the second most common allele in a commonly recognized global population (e.g., 1000 Genomes Project). We refer to this scenario as the augmented SB attack (ASBA).

Formally, the log-likelihood of a set of beacon responses *x* = {*x*_1_, ⋯, *x*_*m*_} and a set of SNVs *d*_*i*_ = {*d*_*i*,1_, ⋯, *d*_*i*,*m*_} for target *i* is:

1 $$ L\left({d}_i, x\right)={\displaystyle {\sum}_{j=1}^m{d}_{i j}}\left({x}_j\kern0.5em  log\kern0.5em  P\left({x}_j=1\right)+\left(1-{x}_j\right)\kern0.5em  log\kern0.5em  P\left({x}_j=0\right)\right) $$

where *d*_*ij*_ and *x*_*j*_ are binary variables. Specifically, *d*_*ij*_ = 1 when SNV *j* of target *i* has at least one alternative allele and *d*_*ij*_ = 0 when SNV *j* of target *i* has no alternative alleles. Additionally, *x*_*j*_ = 1 when SNV *j* has at least one alternative allele in the beacon; *x*_*j*_ = 0 if SNV *j* has no alternative alleles in the beacon.

The null hypothesis can be formulated as:

2 $$ {L}_{H_0}\left({d}_i, x\right)=\kern0.5em {\displaystyle \sum_{j=1}^m{d}_{i j}}\left({x}_j\; log\left(1-{D}_n^j\right)+\left(1-{x}_j\right)\kern0.5em  log\left({D}_n^j\right)\right) $$

And the alternative hypothesis can be formulated as:

3 $$ {L}_{H_1}\left({d}_i, x\right)=\kern0.5em {\displaystyle {\sum}_{j=1}^m{d}_{i j}}\left({x}_j\kern0.5em  log\kern0.5em \left(1-\delta {D}_{n-1}^j\right)+\left(1-{x}_j\right)\kern0.5em  log\kern0.5em \left(\delta {D}_{n-1}^j\right)\right) $$

where *δ* is the sequencing error rate, *D*_*n*_^*j*^ represents the probability that none of the *n* genomes in the beacon have an alternative allele at SNV *j*:

4 $$ {D}_n^j={\left(1-{f}_j\right)}^{2 n} $$

Here, *f*_*j*_ is the AAF of SNV *j* in the population.

The LRT statistic for target *i* can thus be stated as:

5 $$ \begin{array}{l}\Lambda \left({d}_i, x\right)={L}_{H_0}\left({d}_i, x\right)-{L}_{H_1}\left({d}_i, x\right)\\ {}=\kern0.5em {\displaystyle {\sum}_{j=1}^m{d}_{i j}}\left({x}_j\kern0.5em  log\frac{1-{D}_n^j}{1-\delta {D}_{n-1}^j}+\left(1-{x}_j\right)\kern0.5em  log\frac{D_n^j}{\delta {D}_{n-1}^j}\right)\end{array} $$

Given this statistic, a threshold is selected, such that only targeted genomes with a test statistic below the threshold are regarded as being in the beacon. We assume that the attacker will always select the threshold according to a maximal allowable false positive rate (FPR).

**Fig. 1:**
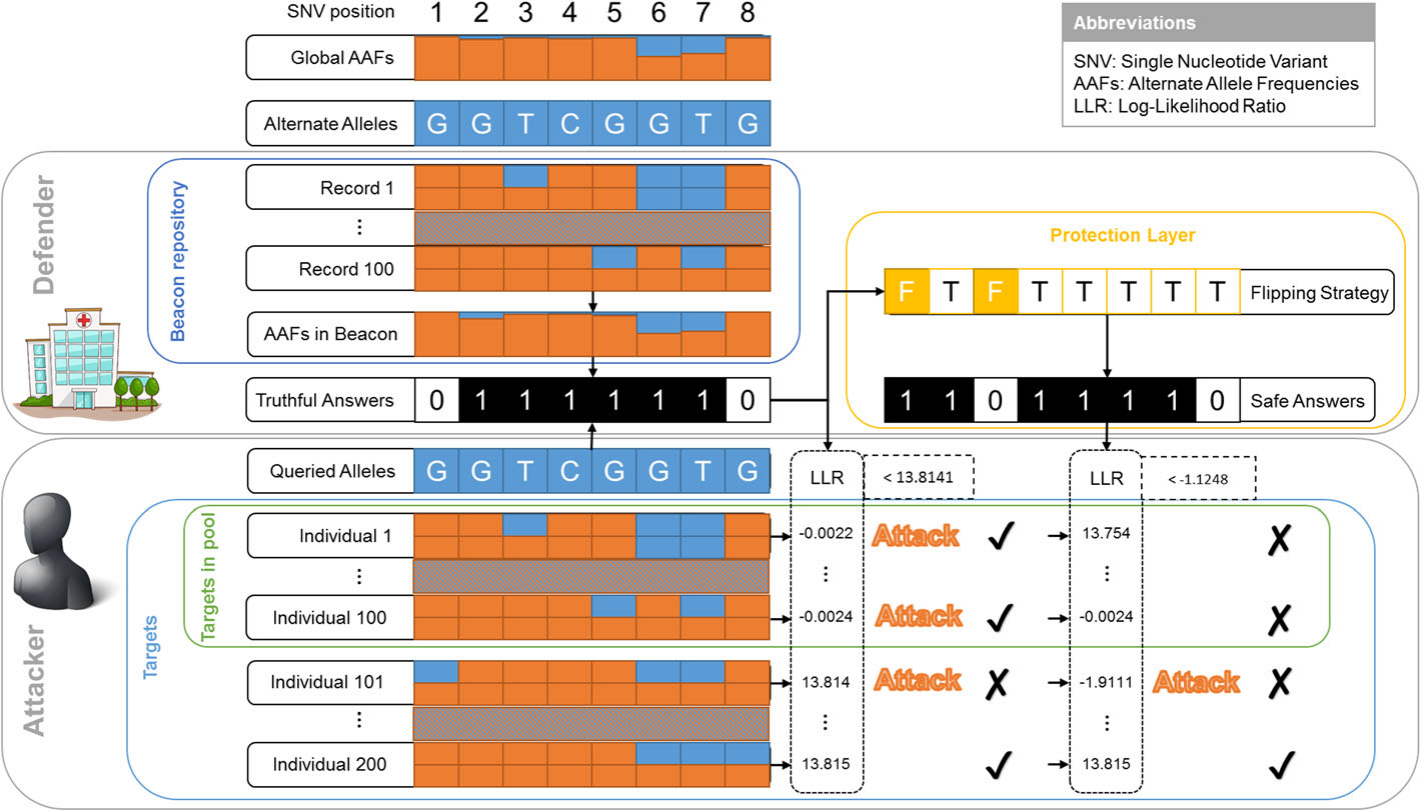
An illustration of the ASBA attack and defense process of the iDASH challenge

To illustrate the ASBA, we present an example of the entire attack and defense process of the iDASH challenge in Fig. 1. For this example, we selected eight SNVs from chromosome 10 and populated a pool of 100 records in a beacon repository according to their global AAFs. Now, let us say the attacker has access to a set of genomes with known identities, which we refer to as a target set. The target set can be divided into two mutually exclusive subsets: i) a set of targets that are actually in the pool and ii) a set of other targets. The attacker will query the Beacon service about whether each alternative allele is in the pool behind the beacon and make their attack decision based on all of the returned answers. If the defender in control of the beacon server answers truthfully, the attacker is likely to achieve a high detection rate. However, if the defender invokes some data protection method (as we introduce later on), then the risk will be mitigated. A flipping action of “T” and “F” for a particular SNV position represents answering the query regarding this position truthfully and untruthfully, respectively. In this example, as shown in the bottom right corner of Fig. 1, it can be seen that the risk has been mitigated substantially.

### Optimization problem

#### Strategies available to the defender

The above problem description implies that the only action the defender can take is to lie in their answers to the attacker’s queries.

In practice, the answer to a query from the attacker for a particular allele in the beacon can be 1, 0 and *null*, the latter of which means that the answer to the query is not applicable (e.g., when the defender does not have any records that cover the SNV of interest to the attacker). Henceforth, for simplicity, and alignment with the data analyzed in the iDASH Challenge, we assume that all data accessible through the beacon is a single nucleotide variant (SNV). In the attack model, the *zero* and *null* answers can be treated differently. The contributions from the SNV *j* to the final LRT statistics according to Equation (5) for answers 1, 0 and *null* are $log\frac{1-{D}_n^j}{1-\delta {D}_{n-1}^j}$, $log\frac{D_n^j}{\delta {D}_{n-1}^j}$, and zero, respectively. Lying about the answer to a query means two alternatives: 1) flipping or 2) masking. We define flipping as changing the answer from 1 → 0 or 0 → 1. We define masking as changing the answer from 1 → *null*, or from 0 → *null*. It should be recognized that we only consider the former type of lies for simplicity in presentation. In other words, we choose to flip each SNV or not. Now, let us say that S_*d*_ = {s_*d*_} is the set of the strategies available to the defender, where each strategy represents a set of SNVs. Then, the number of all available strategies is |*S*_*d*_| = 2^*m*^.

#### Query sequences available to the attacker

The effectiveness of the defender’s strategy is influenced by the attacker’s query sequence. We further assume the attacker has a pre-determined query sequence and has the potential to query all SNVs. Let us say that S_*a*_ = {*s*_*a*_} is the set of query sequences over all of the SNVs available to the attacker. Then the number of all possible query sequences is |*S*_*a*_| = *m* !.

We also assume that the only uncertain action raised by the attacker for the defender is the SNV query sequence. All of the other parameters are fixed and known to the defender before he chooses the best strategy.

#### Objective function

Given this formulation, the iDASH Challenge scenario can be modeled as an optimization problem for the defender. Specifically, we wish to find a set of SNVs to flip that maximizes the utility and the privacy of the data simultaneously.

The effectiveness of a defender’s strategy, *Y*(*s*_*d*_, *s*_*a*_), considering both the utility and the privacy, is a function of both the defender’s strategies and the attacker’s query sequences, which is defined below. This creates a dependency on the definitions of the utility and privacy measures, the manner by which utility and privacy are combined, and the attack model.

As the attacker proceeds through the ordered set of SNVs, he runs a hypothesis test based on the responses for the subset of SNVs queried so far. Now, we assume the defender does not know the ground truth of the attacker’s query sequence. As a consequence, the defender’s best strategy is the one that maximizes his or her own expected effectiveness:

6 $$ {s^{\ast}}_d=\kern0.5em {\mathrm{argmax}}_{s_d} E\left( Y\left({s}_d\right)\right)\kern0.5em =\kern0.5em {\mathrm{argmax}}_{s_d}\frac{1}{\left|{S}_a\right|}\underset{s_a\in {S}_a}{\Sigma} Y\left({s}_d,{s}_a\right) $$

This implies that the attacker will choose any of the query sequences with equal probability. So long as this effectiveness function is defined and known to the defender, the simplest solution for the defender is to examine all available strategies. However, such a brute force approach is computationally challenging because the size of the defender’s strategy space, |*S*_*d*_|, increases exponentially with the number of SNVs.

Similarly, calculating the exact valuation of a defender’s strategy, *E*(*Y*(*s*_*d*_)), is difficult because of the large number of query sequences available to the attacker, |*S*_*a*_|. However, the valuation of a defender’s strategy can be estimated based on a subset of the attacker’s strategies S_*a*_^'^, under a random selection model.

#### Evaluation criteria

There are many alternative definitions of effectiveness that could be invoked to evaluate the defender’s strategies. Here, we first introduce the approach that was relied upon in the iDASH Challenge and then consider several alternatives. As will become evident, this is important because it influences how the defender will search for their best strategy.

In the description of the iDASH Challenge task, the effectiveness of a strategy was defined as the number of answers that can be served correctly before any targeted individual’s presence is successfully detected. However, no matter what the defender’s strategy may be, the detection power of the LLR test is always larger than 0 for the first several SNVs unless queries for these SNVs are not answered truthfully. This implies that at least one individual will be successfully detected if the first several SNVs are answered truthfully, which is highly likely because we assume the defender does not know the position of a specific query in the entire query sequence.

However, such a definition dictates towards a worst-case privacy scenario. Specifically, it assumes that a system is considered vulnerable if any one record can be breached. As noted earlier, there are alternative definitions that could be applied. For instance, a more pragmatic definition of the effectiveness of a protection model may be the proportion of correct answers that are returned before the presence of a certain number of individuals is detected. Under this formulation, when a method is evaluated, the iDASH Challenge organizers stated that 60%, instead of 0%, would be applied as the threshold for the detection power.

To define several alternative evaluation criteria, let us limit the utility and privacy measures in the [0, 1] range. For the purposes of the iDASH Challenge, utility can be regarded as the proportion of queries that are answered truthfully. By contrast, privacy is defined as a binary variable that is one if, and only if, a certain portion of the targeted individuals are never detected in the beacon, and 0 otherwise. Now, there are numerous ways to combine the utility and privacy measures into an effectiveness measure. In the iDASH Challenge, the effectiveness of the defense was defined as the utility for the proportion of SNVs shared before a certain portion of targeted individuals are re-identified.

Alternatively, the utility can be defined as a weighted sum of correct answers. In this scenario, each SNV can be weighted according to its importance (e.g., correlation with some phenotype). On the other hand, privacy can be defined as the expected false negative rate when the number of used SNVs is uncertain. The effectiveness of the defense can thus be defined as a weighted sum of the utility and the privacy.

### Protection method

In this section, we start with a description of the solution we submitted to the iDASH Challenge. To perform a comprehensive empirical analysis, we then provide a description of alternative methods that could be applied to this problem.

### Our iDASH submission

The solution we submitted to the iDASH Challenge entails searching through a collection of possible strategies that the defender can invoke to protect the system. Each of these strategies utilizes the same method, in the form of flipping some SNV query answers. In this section, we illustrate the principles by which such answers are flipped and how the strategy space is prioritized and searched.

#### Flipping responses

In the iDASH Challenge, it was assumed that the defender is not aware of the attacker’s query sequence a priori and does not keep track of the queries. As a result, we need to find a defender’s strategy that is independent of the attacker’s query sequence. Since utility is basically measured as the number of queries that the defender responds to truthfully, we introduce the notion of discriminative power for each SNV in the pool. The *discriminative power* represents an SNV’s ability to distinguish the records in the pool of individuals behind the beacon from a reference dataset. We define a *differential discriminative power* for each SNV in the pool, which represents the difference between its discriminative power before and after a flip. The top $k$ percent of the SNVs in the pool, ranked by their differential discriminative power, will have their query responses flipped.

Here, we take a moment to formally define the discriminative power. We assume that the defender knows the attacker’s target set (because, otherwise, the defender’s strategy could not be directly measured). If this is not the case, then there are various ways for the defender to estimate the target set (the details of which are deferred to below). Let us say that the targeted pool with $n$ individuals and $m$ SNVs is represented as a binary matrix with *n* rows and *m* columns *D*_*n* × *m*_ = {*d*_*ij*_} in which *d*_*ij*_ is one when individual *i* in the pool has the alternative allele for SNV *j* and zero otherwise. Let us further say that the targeted reference dataset with *n* ^'^ individuals and $m$ SNVs (the same as the SNVs in the pool) is represented by a binary matrix with *n* ^'^ rows and *m* columns ${R}_{n^{\hbox{'}}\times m} = \left\{{r}_{ij}\right\}$ in which *r*_*ij*_ is one when individual $i$ in the reference has the alternative allele in SNV *j* and zero otherwise.

Given the truthful answer *x*_*j*_ for the query regarding SNV *j*, the ability for SNV *j* to indicate if an individual is behind the beacon, according to the pool data, is defined as the average LLR for all the individuals in the pool if only SNV *j* is queried:

7 $$ {A}_j\left({x}_j\right)=\frac{1}{n}{\displaystyle \sum_{i=1}^n{d}_{i j}}\left({L^{\hbox{'}}}_{H_1}\left({x}_j\right)-{L^{\hbox{'}}}_{H_0}\left({x}_j\right)\right) $$

where

8 $$ {L^{\hbox{'}}}_{H_0}\left({x}_j\right)={x}_j\kern0.5em  log\left(1-{D}_n^j\right)\kern0.5em +\kern0.5em \left(1-{x}_j\right)\kern0.5em  log\left({D}_n^j\right) $$

9 $$ {L^{\hbox{'}}}_{H_1}\left({x}_j\right)={x}_j\kern0.5em  log\left(1-\delta {D}_{n-1}^j\right)\kern0.5em +\kern0.5em \left(1-{x}_j\right)\kern0.5em  log\left(\delta {D}_{n-1}^j\right) $$

Similarly, the ability for an SNV to indicate if an individual is behind the beacon, according to the reference dataset, is defined as the average LLR for all the individuals in the reference if only SNV *j* is queried:

10 $$ {A^{\hbox{'}}}_j\left({x}_j\right)=\frac{1}{n^{\hbox{'}}}{\displaystyle \sum_{i=1}^{n\hbox{'}}{r}_{i j}}\left({L^{\hbox{'}}}_{H_1}\left({x}_j\right)-{L^{\hbox{'}}}_{H_0}\left({x}_j\right)\right) $$

The more similar these two values, the less powerful the LLR test will be. Based on this formulation, the discriminative power for SNV *j* becomes:

11 $$ {D}_j\left({x}_j\right)={A}_j\left({x}_j\right)-{A^{\hbox{'}}}_j\left({x}_j\right)=\left(\frac{1}{n}{\displaystyle \sum_{i=1}^n{d}_{i j}}\kern0.5em -\frac{1}{n^{\hbox{'}}}{\displaystyle \sum_{i=1}^{n^{\hbox{'}}}{r}_{i j}}\right)\;\left({L^{\hbox{'}}}_{H_1}\left({x}_j\right)-{L^{\hbox{'}}}_{H_0}\left({x}_j\right)\right) $$

The difference of the discriminative powers before and after flipping the SNV is:

12 $$ \Delta {D}_j\left({x}_j\right)={D}_j\left({x}_j\right)-{D}_j\left(1-{x}_j\right) $$

As a result, the first step of our Strategic Flipping method will flip the top *k* percent of the SNVs sorted according to the differential discriminative power. Note that, when a set of SNVs have the same differential discriminative power, the SNVs with the highest discriminative powers are selected first. When a set of SNVs have the same discriminative power, the SNVs with the lowest AAFs are selected first. A random selection is applied to break AAF ties.

#### Searching the strategy space

We use a greedy algorithm to search the defender’s strategy space for a local optimum. To do so, we begin by randomly selecting *q* query sequences. Next, we traverse the *l* nearest neighbors of the current best strategy and find the neighbor with the best average measure of effectiveness, which is averaged across the $q$ query sequences. Two strategies are regarded as neighbors if they only have one answer that is different. The distance between two neighbors is calculated as the absolute difference of the average number of answers provided truthfully by the two strategies and the rank of the different SNVs of these two strategies, in descending order, sorted by the differential discriminative power. In other words, if the number of the answers provided truthfully by these two strategy is *t* and *t* ^'^ and the rank of the different SNV is τ, then the distance between two neighbors is |τ − (*t* + *t* ^'^)/2|. When *l* equals to two, only the top SNVs, in terms of differential discriminative power, are flipped.

We start from the result of the aforementioned Top-K Flipping step and keep searching until no strategy with better effectiveness can be found. In the case where two measures need to be optimized simultaneously (such as utility and privacy measures), we search the neighborhoods for a Pareto-optimal strategy. In a multiple-objective optimization problem, a strategy is Pareto optimal if no other strategies dominate it (i.e., is better than it in terms of both measures in consideration).

### Alternative methods

We selected five alternative methods beyond the one we proposed above. The first three are baseline methods that set the upper and lower bounds on the measures. The last two are state-of-the-art methods in the literature that address the beacon detection problem. For reference purposes, we name our iDASH solution as the Strategic Flipping Method or *M*_*SF*_.

1. **Truthful Method (marked as** ***M***_***T***_ **).** The defender simply responds to all queries truthfully. This sets the lower bound for the privacy measure and the upper bound for the utility measure.
2. **Baseline Method (marked as** ***M***_***B***_ **).** The defender flips the *k* percent of the SNVs with the lowest AAF in the underlying population from which the pool is sampled. This method was used by the organizers of the iDASH Challenge to establish a lower bound for the effectiveness measure.
3. **Greedy Accountable Method (marked as** ***M***_***GA***_ **).** We assume the users’ queries are accountable, whereby each user has an account such that the defender documents the attacker’s queries and results as they are processed. Upon submission of the next SNV query, the defender will flip the answer for this SNV if, and only if, the power of the resulting LLR test would be smaller than when no flip is applied.
4. **Random Flips (marked as** ***M***_***RF***_ **).** This method was recently proposed by Raisaro et al. [32]. In this method, the defender flips ε portion of SNVs that exhibit unique alleles in the beacon.
5. **Query Budget (marked as** ***M***_***QB***_ **).** This method was also proposed by Raisaro et al. [32]. In this method, a privacy budget is assigned to each individual in the beacon. Each time a record contains the queried allele, the budget for that user is reduced by a certain amount. Once a record’s budget is exhausted, their genome will no longer contribute to responses provided by the beacon. Similar to *M*_*R*_, this method requires the users’ queries to be documented.

## Results

In this section, we introduce the evaluation measures, the experimental setup, and the results of the empirical analysis.

### Evaluation measures

In addition to the measure of effectiveness provided by the organizers of the iDASH Challenge, we propose several alternatives to assess the effectiveness of each method in a more comprehensive manner.

### Utility

We measure utility according to the proportion of queries responded to truthfully (*U*).

### Privacy

We measure privacy according to two different criteria, which we refer to as *P*_1_ and *P*_2_. First, we measure *P*_1_ as a binary variable that is set to one if, and only if, a certain portion of the targeted individuals were never detected in the beacon, and 0 otherwise. We select 60% as the threshold because this is the definition used in the iDASH Challenge. It should be noted that our method generalizes to any threshold, but the results we present are limited to this parameterization. Second, we measure *P*_2_ as the expected false negative rate when the number of SNVs to be queried is uncertain. We assume that the total number of SNVs about which the attacker has already queried, when they stop, is a random integer number uniformly distributed in the range [0, m].

### Effectiveness

The effectiveness, which considers both utility and privacy, is measured according to two criteria. First, we measure *E*_1_ as the proportion (in terms of all SNVs) of queries that are responded to truthfully before 60% of the individuals are successfully detected. Second, we measure *E*_2_ as the proportion of truthful answers plus the expected false negative rate.

### Computational efficiency

Computational efficiency is an important factor to be considered for deployment of solutions in a working system of beacons. As such, we also present the running time of each method. To measure the running time, we use a machine with Intel Core i7 quad-core 3.00GHz CPU and 8 GB memory.

### Experimental design

To evaluate the effectiveness of the methods, we created a pool based on the first 400,000 SNVs in Chromosome 10. The pool is composed of 250 individuals randomly selected from the 2,504 individuals in Phase 3 of the 1000 Genomes Project [12]. The reference includes 250 individuals randomly selected from the remaining individuals in Phase 3 of the project. Note that the higher the association between the allele frequencies in the pool and the allele frequencies estimated by the attacker (as demonstrated by a sensitivity analysis), the higher the detection power. In our experiments, we assume a scenario where the allele frequency estimates available to the attacker are the allele frequencies in the entire population of 1000 Genomes. All of the other parameters are set as shown in Table 1. The maximal allowable false positive rate (α) is set to 0.05 and the sequencing error rate (δ or the mismatch rate) is set to 0.000001 according to Shringarpure and Bustamante [30]. The number of sampled query sequences is set to 10, and the number of examined neighbors is set to two for simplicity. The percentage of flipped answers is set to five, but this setting will be discussed further in the section of sensitivity analysis. The noise level in the Random Flip method is set to 0.75, and the maximal allowable power in the Query Budget method is set to 0.9 according to Raisaro et al. [32].

**Table 1:** Parameter settings for the experiments

Parameter	Notation	Setting
Size of pool	*n*	250
Number of SNVs	*m*	400,000
Maximal allowable false positive rate	α	0.05
Sequencing error rate	*δ*	0.000001
Number of sampled query sequences	*q*	5
Number of examined neighbors	*l*	2
Percentage of flipped answers	*k*	5
Noise level in the Random Flip method	ε	0.75
Maximal allowable power in the query budget method	β	0.9

### Findings

**Fig. 2:**
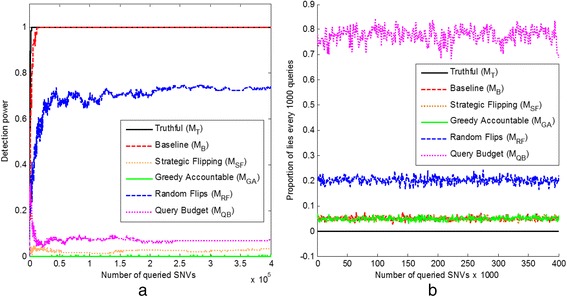
How the number of SNVs queried influences the (**a**) detection power and (**b**) proportion of lies

We compare all methods using the average results across 10 randomly generated query sequences. Fig. 2 shows how the detection power and the proportion of flipped answers (i.e., lies to the attacker) every 1000 queries change as a function of the number of queried SNVs for one of the query sequences.

By inspecting Fig. 2(a) and (b), it can be seen that the detection power does not increase monotonically when the defender is flipping answers. From Fig. 2(a), it can be seen that the three methods with the lowest (on average) detection power are *M*_*GA*_, *M*_*SF*_, and *M*_*QB*_. Notably, none of these methods exceed the threshold of 60%. From Fig. 2(b), it can be seen that the three methods with fewest (on average) induced lies are *M*_*GA*_, *M*_*B*_, and *M*_*T*_. Considering the intersection of these results, it appears that *M*_*RF*_ and *M*_*GA*_ are likely the best options.

**Fig. 3:**
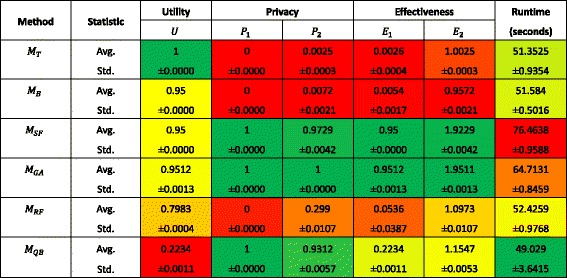
The performance of the genomic data protection methods across 10 runs

Inspecting the result of only one query sequence provides some intuition into the trends of the utility and privacy measures, but it may be biased by a single run of the experiment. Fig. 3 summarizes the mean and +/-1 standard deviation for each of the performance measures across 10 query sequences.

Figure 3 reveals several notable findings. First, it can be seen that, according to the evaluation measures defined in the iDASH Challenge (*E*_1_), our proposed method (*M*_*SF*_) is the second best. However, since the best method (*M*_*GA*_) assumes the users are accountable - which does not exist in the current system - it cannot be regarded as a practical solution for the iDASH Challenge.

With respect to effectiveness, we find that the two measures ( *E*_1_ and *E*_2_) are in complete agreement regarding the rank order of the best methods. With respect to privacy, the second measure (*P*_2_) does a better job of distinguishing between the methods than the first measure.

With respect to running time, we find that the solution we proposed ( *M*_*SF*_) exhibited the longest running time - about 56% longer than the quickest method. However, we assume that such a difference efficiency may not be critical because the defender’s strategy for each beacon is determined once the beacon is published rather than calculated on-the-fly.

**Fig. 4:**
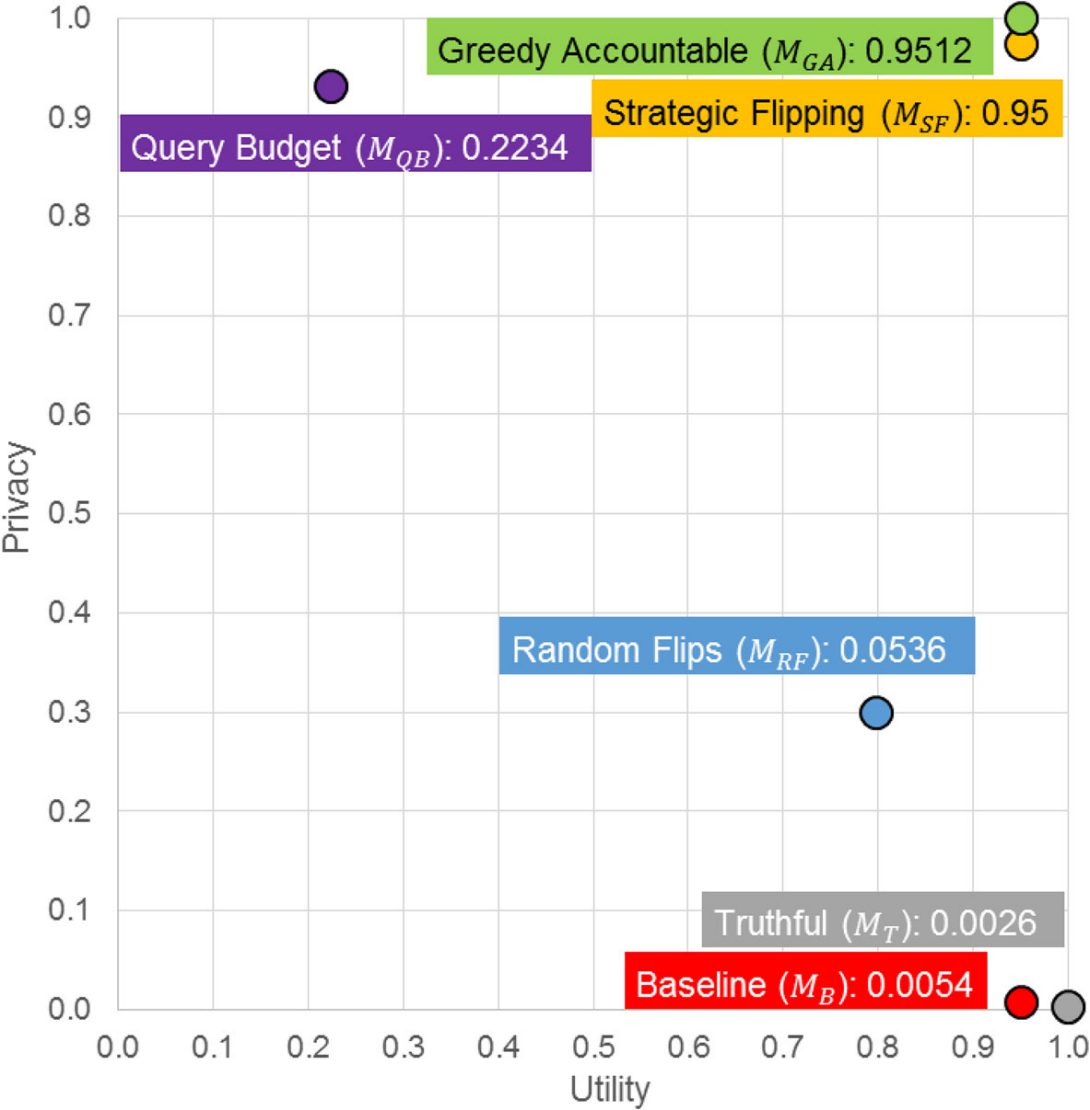
A comparison of the genomic data protection methods with respect to utility and privacy

While there is no perfect way to combine the utility and privacy measures, we can compare the methods directly on these dimensions. In Fig. 4, for each method, utility ( *U*) is shown by the x-axis, privacy ( *P*_2_) is shown by the y-axis, and effectiveness (*E*_1_) is shown in text. It can be seen that, after dismissing the two impractical methods (*M*_*T*_ and *M*_*GA*_), the method we proposed for the iDASH Challenge dominates all other solutions.

### Sensitivity Analysis

To gain a deeper appreciation for the stability of the results of the iDASH Challenge, we assessed the performance of the proposed methods when certain key parameters are varied.

### Tunable parameters

All of the protection methods, except *M*_*T*_ and *M*_*GA*_, include a tunable parameter. Here, we systematically investigate how changes to the value of this parameter influences their performance. For brevity in presentation, we designed four cases: two with values smaller and two with values larger than the value applied in the scenario investigated above. The specific values for the sensitivity analysis are detailed in Table 2. Only the most representative values are chosen for each parameter. The value of each parameter in Case three (Mid) is the default value as we used in the above experiment that simulated the iDASH Challenge. The values for each parameter in Case one (Low) and Case five (High) are the smallest and largest values, respectively, while Case two (Low-Mid) and Case four (Mid-High) provide gradations between these case to provide a more complete view.

**Table 2:** Parameterizations for the sensitivity analysis of the genomic data protection methods

Method	Parameter	Case 1 (Low)	Case 2 (Low-Mid)	Case 3 (Mid)	Case 4 (Mid-High)	Case 5 (High)
*M*_*B*_	Percentage of flipped answers (*k*)	1	2	5	10	20
*M*_*SF*_						
*M*_*RF*_	Noise level (ε)	0.1	0.5	0.75	0.9	1
*M*_*QB*_	Maximal allowable power (β)	0.1	0.5	0.9	0.95	1

**Fig. 5:**
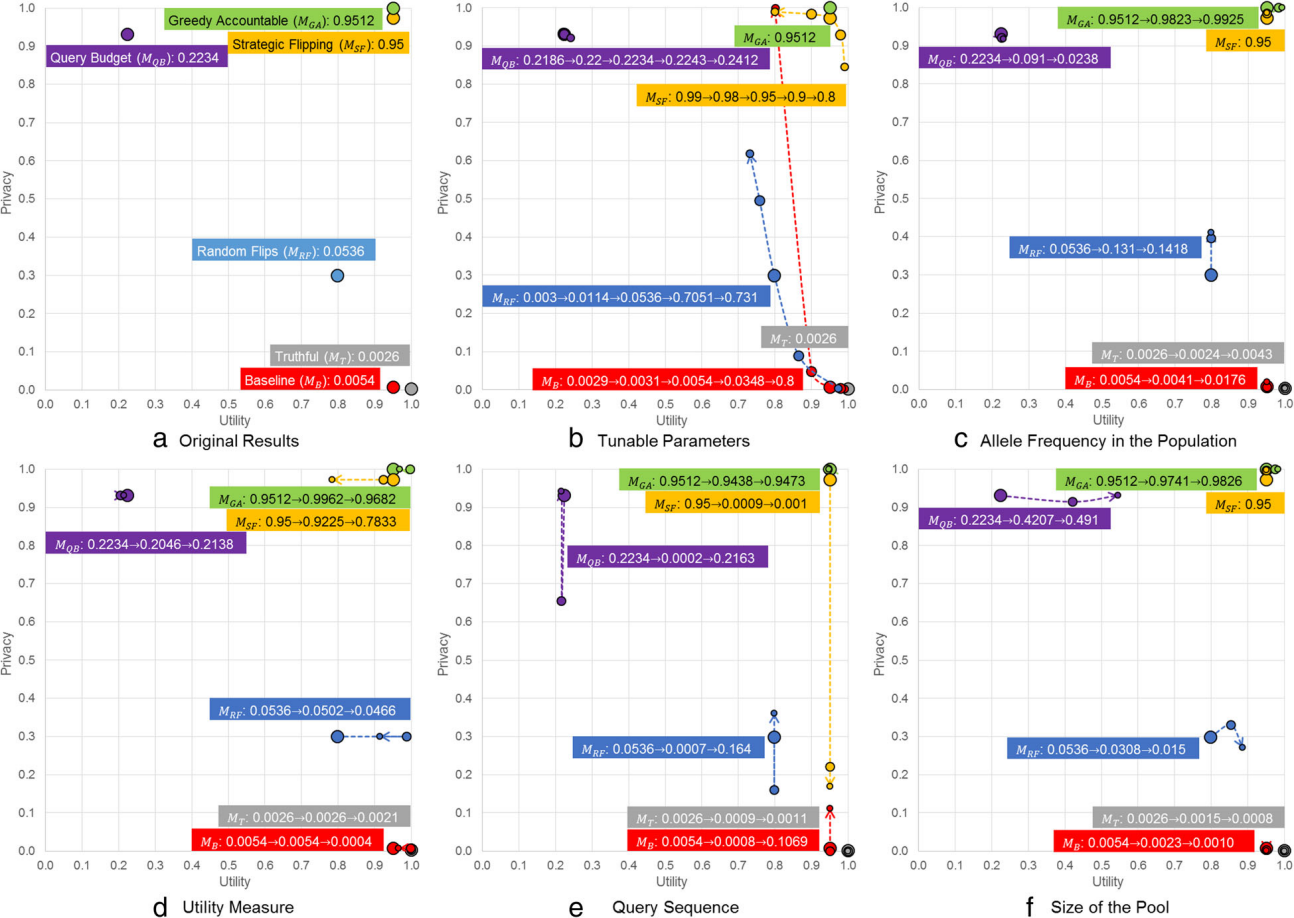
Influence of the key parameters and factors on the utility and privacy measures. **a** Original Results. **b** Tunable parameters. **c** Attacker’s knowledge about allele frequencies in the population. **d** The utility measure. **e** Query sequence. **f** Size of the Pool

Figure 5(b) displays the results of the sensitivity analysis with respect to the utility, privacy and effectiveness measures. The series of numbers near the series of circles represent the effectiveness (*E*_1_) of the methods in different cases (from the Low Case to the High Case). There are several notable findings from this analysis to highlight. First, it should be recognized that utility is negatively correlated with the parameter in methods *M*_*B*_, *M*_*SF*_ and *M*_*RF*_ and positively correlated with the parameter in the method *M*_*QB*_. Second, privacy (*P*_2_) is positively correlated with the parameter in *M*_*B*_, *M*_*SF*_ and *M*_*RF*_ and negatively correlated with the parameter in *M*_*QB*_. Third, effectiveness (*E*_1_) is positively correlated with the parameter in *M*_*B*_, *M*_*RF*_ and *M*_*QB*_ and negatively correlated with the parameter in the *M*_*SF*_. The effectiveness of our proposed *M*_*SF*_ method is larger than all of the alternative methods, including *M*_*GA*_, when the value for the *k* parameter is smaller than five. Most importantly, we found that the method we proposed for the iDASH Challenge dominates all of the alternative methods - except *M*_*T*_ and *M*_*GA*_ when the value for the *k* parameter is two.

### Allele frequency in the population

In the iDASH Challenge, it was assumed that the attacker’s estimate of the allele frequencies is similar to those in the 1000 Genomes population of around 2500 individuals. However, in the real world, the attacker is likely to have a stronger capability. For instance, the attacker may know the allele frequencies are from a smaller population.

Thus, we investigated how an enhancement of the attacker’s capability influences the performance of the methods. Specifically, we assess the performance of the protection methods when the attacker has a more accurate estimate of the allele frequencies by gaining access to a smaller population of only 500 individuals. We further examine the scenario where the attacker relies on allele frequencies that are exactly the same as the pool behind the beacon.

Figure 5(c) depicts the utility, privacy and effectiveness measures of different methods. The series of numbers near the series of circles (from larger to smaller circles) represent the effectiveness (*E*_1_) of the methods in different cases (from larger to smaller populations). There are several important take-away messages from this analysis. First, both the effectiveness of protection (in terms of both *E*_1_ and *E*_2_) increases for *M*_*B*_, *M*_*SF*_, *M*_*GA*_, *M*_*RF*_ and decreases for *M*_*QB*_ when the attacker’s allele frequency estimate becomes more accurate. Second, the solution we proposed for the iDASH Challenge remains the best method for current application (noting that *M*_*GA*_ still remains the best overall).

### Utility measure

In the iDASH Challenge, we assumed the utility for each SNV was equivalent. Here, we assume the utility is measured as a weighted sum of the truthful answers. The weight for each SNV can be (1) the absolute difference between the allele frequency in the pool and population or (2) a worst-case scenario for our proposed method, where the utility is equal to the absolute differential discriminative power the defender used for each SNV.

The results of this analysis are shown in Fig. 5(d). The series of numbers near the series of circles (from larger to smaller circles) represent the effectiveness (*E*_1_) of methods in different cases (from the original setting to the Low Case, and from the Low Case to the Low-Mid Case). In these scenarios, we find that our proposed *M*_*SF*_ for the iDASH Challenge performs substantially worse than it did in the challenge because utility is measured differently. Nonetheless, even in such an extreme case, our method is never dominated by others, except *M*_*GA*_, and dominates *M*_*QB*_. However, it should be recognized when method *A* fails to dominate method *B*, it does not imply that method *B* dominates method *A*.

### Query sequence

Different sequences of the queries have the potential to yield different detection results. As such, we need to consider how well the attacker can perform if he chooses the sequence with the highest possible detection power. Let us consider two scenarios: (1) the attacker always queries the most discriminative set of SNVs first; (2) the attacker always queries the rarest SNVs first.

The performance of the protection methods with respect to these two scenarios are depicted in Fig. 5(e). The series of numbers near the series of circles (from larger to smaller circles) represent effectiveness (*E*_1_) of the methods in different cases (from the original setting to case 1, and from case 1 to case 2). As anticipated, it can be seen that the defender tends to lose privacy no matter what protection method is invoked in the first scenario. This is because the SNVs queried first have very strong discriminative power. However, in the second scenario, the defender loses privacy only when our proposed method is invoked and gains privacy when other methods are invoked.

These results primarily stem from three reasons. First, the SNVs with high differential discriminative power are not the same as the SNVs with high discriminative power or the SNVs with low alternative allele frequency. As a result, in the face of these two query sequences, the defender does not flip any SNVs until the very end of the query sequence. This leads to a high detection power quickly. Second, a flipping strategy works best in the scenario where all SNVs that need to be flipped are also queried first. In other words, our proposed method works best when the top $k$ percent SNVs with highest differential discriminative power are queried first. By contrast, the baseline method works best when the top $k$ percent SNVs with lowest alternative allele frequency are queried first. Third, Raisaro et al. [32] assumed that the rarest SNVs are queried first. Thus, the Random Flips method (*M*_*RF*_) and the Query Budget method (*M*_*QB*_) perform well in the scenario where this assumption holds true. Still, the solution we proposed for the iDASH Challenge is not dominated by any of the other methods except for the Greedy Accountable method (*M*_*GA*_) and dominates the baseline method (*M*_*B*_). Still, if the attacker’s query sequence is fixed and known by the defender, the Greedy Accountable method (*M*_*GA*_) becomes practical and the defender will end up with nearly perfect scores.

### Size of the pool

In the iDASH Challenge, we used a dataset where there were 250 individuals in the pool behind the beacon. Here, we consider scenarios where there are fewer individuals in the beacon. Specifically, we assess the performance of the methods when there are only 1) 100 individuals and 2) 50 individuals in the pool. The results are shown in Fig. 5(f). The series of numbers near the series of circles (from larger to smaller circles) represent the effectiveness (*E*_1_) of the protection methods in different cases (from larger to smaller sized pools).

It can be seen that the effectiveness of protection is positively correlated with the pool size in methods *M*_*T*_, *M*_*B*_ and *M*_*RF*_. And, once again, the method we submitted to the iDASH Challenge remains the best practical method in terms of all evaluation measures.

## Discussion

The findings illustrate that the ASBA attack against a Beacon service can be sufficiently mitigated through a strategy that intelligently prioritizes which genomic variants to provide truthful answers about. However, we wish to point out that addressing the iDASH Challenge task is only the first step to solving the more general problem of mitigating the SB attack against the Beacon service in the real world. To create a more realistic solution, we wish to highlight two sets of limitations. The first set is an artifact of the design of the iDASH Challenge, while the second set is an artifact of the design of our model.

### Limitations of the iDASH Challenge

There are, at least, three key limitations to the design of the iDASH challenge that hinder its practicality: 1) the evaluation measure, 2) the available strategy space, and 3) the construction of the attack model.

First, the iDASH Challenge regarded each SNV as having the same utility. This is critical to recognize because all of the defender’s strategies trade between utility and privacy. In our proposed method, we first flip answers for the SNVs that will lead to the greatest loss of privacy. Yet, if these SNVs happen to have a more severe influence on utility than other SNVs, the best strategy for the defender will likely change.

Second, the Beacon service in the iDASH Challenge allows the defender to flip an answer from zero to one or from one to zero when answering the queries from a user. However, in the real world, the defender could have access to either a smaller or a larger strategy space. For instance, a smaller strategy space might be realized if a data custodian only allows for flipping from one (presence of a variant) to zero (absence of a variant) - in other words, the data custodian chooses to never lie about presence. By contrast, a larger strategy space might be created if an data custodian can play out their actions over a series of stages to query response. For example, as Raisaro et al. mentioned [24], if each user must create an account to query the beacons, the data custodian can distinguish between them and document their query history. As a consequence, the Query Budget (*M*_*QB*_) and Greedy Accountable (*M*_*GA*_) and methods will become practical, while the latter could become the optimal solution.

Still, the strategy space for the defender is likely to be much larger than what we have alluded to because they may have access to more than technical methods to lean on for protection. For instance, the defender may institute legal and/or economic methods to change the utility of the system for the attacker. For example, if each user must sign a data use agreement before querying a Beacon service, the malicious users might be detected as attempting to re-identify records and could be pursued as violators of a contract and penalized for liquidated damages (e.g., sued for a monetary loss). Note that such approaches will add minimal burden on typical law abiding users, but could come at a substantial cost for the defender. Still, in this scenario, a cost-benefit analysis becomes necessary for an economically-motivated defender, such that the defender will need to choose the strategy that optimizes his monetary payoff while satisfying the privacy requirements. And, when the attacker makes a decision based upon the defender’s strategy, a game theoretic analysis should be invoked to solve this problem as shown by Wan et al. [33].

Third, both the original and augmented SB attack in the iDASH Challenge consider an adversarial model that tends to overestimate the privacy risk. This happens for several reasons. First, the attacker does not know the ground truth about who is in the pool. As such, he does not know the exact false positive rate. When he overestimates the false positive rate, fewer individuals will be attacked. Second, as pointed out by Craig and colleagues [25] the prior probability that a targeted individual is actually in the pool is likely to be much smaller than 50% as was the case for the iDASH Challenge, which can substantially influence the statistical inference power [33]. Third, it should be recognized that a possible attack is not the same as a probable attack. When an attacker is economically-motivated, he is unlikely to attack when the cost exceeds the benefit of an attack.

### Limitations of the protection method

There are three primary aspects of our protection method that should be addressed before it is instituted in practice: 1) the deterministic approach to flips, 2) an estimation of the attacker’s target set, and 3) a grounded approach to select *k* (i.e., the percentage of SNVs to flip).

First, our solution invokes a deterministic approach to selecting which SNVs to flip. This is potentially problematic because, if the attacker was able to ascertain some of the allele frequencies in the pool behind the beacon, then they could mimic the strategy of the defender. In other words, the attacker would be able to determine which SNVs the defender would choose to lie about. As a consequence, each query response for such SNVs could then be flipped back to the correct answer about the underlying pool, thus rescinding all of the protection. Therefore, in the event that there is a concern about such exposure, our model could incorporate a randomization component, where the answers provided to the adversary are non-deterministic. If such a feature were to be incorporated, it is critical to minimize the level of randomization to achieve the desired level of security.

Second, in the iDASH Challenge, the target set (i.e., the set of genomic records for presence/absence testing) was provided to the competition teams. However, in the real world, there may be multiple attackers, each of which may harbor a different target set. In such a scenario, the computation of the discriminative power for each SNV in the pool should be dependent on the underlying population of the beacon instead of a particular target set. In other words, when the defender is uncertain about the number of attackers and their targets, the entire population from which the beacon is sampled from should be used as the target set in our model. However, the mismatch in target sets may affect the performance of our method.

Finally, the parameter *k* in our method determines the starting point of the search for local optimal strategy. A well-specified value of *k* increases the probability that the local optimal strategy is also globally optimal. The best choice for *k* is dependent upon a number of factors, including 1) the size of the pool, 2) the number of SNVs, 3) the maximal allowable false-positive rate, 4) the specific data in the pool, and 5) the target set. In practice, when the defender needs to determine *k*, he could simulate an attacker with an estimated maximal allowable false-positive rate (which is often set to 5%), as well as a target set, and then select the best choice empirically. For example, according to the results in Fig. 4, the best choice of *k* is five in terms of maximizing the effectiveness (*E*_2_).

## Conclusions

This paper introduced a technical solution for mitigating the Shringapure and Bustamante (SB) attack on the Beacon service of the Global Alliance for Genomics and Health. This solution was specifically tailored to address an augmented version of this attack as posed in the 2016 iDASH Challenge. This solution was the winning entry and in this paper, we provided a formalization of the iDASH Challenge problem, a general design of the protection model, and an empirical evaluation of our solution to demonstrate its potential for protecting privacy with minimal influence on the utility of the system within a practical computational runtime.

We further showed, via an extensive experimental evaluation, that our proposed method outperforms all posited baseline and state-of-the-art methods (that were applicable to real world scenarios) regardless of how key parameters that drive the attack (e.g., the effectiveness measure, the number of records behind the beacon, and the attacker’s estimate of allele frequency) vary. In most scenarios, the advantages of our method over other alternative methods are substantial. Still, it should be recognized that our method is limited by the design of the iDASH Challenge scenario (e.g., a strategy space limited to changing query answers) and the evaluation protocol (e.g., adversarial knowledge of minor allele frequencies).

## Abbreviations

AAF: Alternative allele frequency
ASBA: Augmented SB attack
dbGaP: Database of Genotypes and Phenotypes
FPR: False positive rate
GA4GH: Global Alliance for Genomics and Health
iDASH: Integrating Data for Analysis, Anonymization, and Sharing
LLR: Log-likelihood ratio
LRT: Likelihood ratio test
SB: Shringarpure and Bustamante
SNV: Single nucleotide variant

## References

[1] Stephens ZD, Lee SY, Faghri F, Campbell RH, Zhai C, Efron MJ, Iyer R, Schatz MC, Sinha S, Robinson GE (2015). Big data: astronomical or genomical?. PLoS Biol.

[2] Philips AM (2016). Only a click away – DTC genetics for ancestry, health, love… more: a view of the business and regulatory landscape. Appl Transl Genomics.

[3] Rehm HL (2013). Disease-targeted sequencing: a cornerstone in the clinic. Nat Rev Genet.

[4] Taber KAJ, Dickinson BD, Wilson M (2014). The promise and challenges of next-generation genome sequencing for clinical care. JAMA Intern Med.

[5] Green ED, Guyer MS, and the National Human Genome Research Institute (2011). Charting a course for genomic medicine from base pairs to bedside. Nature.

[6] Gottesman O, Kuivaniemi H, Tromp G (2013). The electronic medical records and genomics (eMERGE) network: past, present and future. Genet Med.

[7] Collins F, Varmus H (2015). A new initiative on precision medicine. N Engl J Med.

[8] Aronson SJ, Rehm HL (2015). Building the foundation for genomics in precision medicine. Nature.

[9] Boycott KM, Vanstone MR, Bulman DE, MacKenzie AE (2013). Rare-disease genetics in the era of next-generation sequencing: discovery to translation. Nat Rev Genet.

[10] Kobalt DC, Steinberg KM, Larson DE, Wilson RK, Mardis ER (2013). The next-generation sequencing revolution and its impact on genomics. Cell.

[11] ACMG Board of Directors. Laboratory and clinical genomic data sharing is crucial to improving genetic health care: a position statement of the American College of Medical Genetics and Genomics. Genetics in Medicine. 2017; doi:10.1038/gim.2016.196. 10.1038/gim.2016.196 28055021

[12] Hayden EC (2013). Geneticists push for global data-sharing. Nature.

[13] Ball MP, Bobe JR, Chou MF, Clegg T, Estep P, Lunshof JE, Vandewege W, Zaranek AW, Church GM (2014). Harvard personal genome project: lessons from participatory public research. Genome Med.

[14] Sanderson SC, Linderman MD, Suckiel SA, Diaz GA, Zinberg RE, Ferryman K, Wasserstein M, Kasarskis A, Schadt EE (2016). Motivations, concerns and preferences of personal genome sequencing research participants: baseline findings from the HealthSeq project. Eur J Hum Genet.

[15] Hull SC, Sharp RR, Botkin JR, Brown M, Hughes M, Sugarman J, Bolcic-Jankovic D, Clarridge BR, Wilfond BS (2008). Patients views on identifiability of samples and informed consent for genetic research. Am J Bioeth.

[16] Kaufman DJ, Muphy-Bollinger J, Scott J, Hudson K (2009). Public opinion about the importance of privacy in biobank research. Am J Hum Genet.

[17] Mailman MD, Feolo M, Jin Y, Kimura M, Tryka K (2007). The NCBI dbGaP database of genotype and phenotypes. Nat Genet.

[18] Homer N, Szelinger S, Redman M, Duggan D, Tembe W (2008). Resolving individuals contributing trace amounts of DNA to highly complex mixtures using high-density SNV genotyping microarrays. PLoS Genet.

[19] Frazer KA, Ballinger DG, Cox DR, Hinds DA, International HalMap Consortium (2007). A second generation human haplotype map of over 3.1 million SNVs. Nature.

[20] Auton A, Brooks LD, Durbin RM, Garrison EP, Kang HM, Korbel JO, Marchini JL, McCarthy S, McVean GA, Abecasis GR, And 1000 Genomes Project Consortium (2015). A global reference for human genetic variation. Nature.

[21] Felch J. DNA profiles blocked from public access. Los Angeles Times. August 29, 2008. URL: http://articles.latimes.com/2008/aug/29/local/me-dna29. Accessed 4 June 2017.

[22] Zerhouni EA, Nabel EG (2008). Protecting aggregate genomic data. Science.

[23] Sankararaman S, Obozinski G, Jordan MI, Halperin E (2009). Genomic privacy and limits of individual detection in a pool. Nat Genet.

[24] Wang R, Li YF, Wang XF, Tang H, Zhou W. Learning your identity and disease from research papers: information leaks in genome wide association study. Proceedings of the 16th ACM Conference on Computer and Communications Security. 2009: pp. 534-44. http://dx.doi.org/10.1145/1653662.1653726.

[25] Craig D, Goor RM, Wang Z, Paschall J, Ostell J, Feolo M, Sherry ST, Manolio T (2011). Assessing and managing risk when sharing aggregate genetic variant data. Nat Rev Genet.

[26] Gymrek M, MCGuire A, Golan D, Halperin EE (2013). Identifying personal genomics by surname inference. Science.

[27] Dwork C, Smith A, Steinke T, Ullman J, Vadhan S. Robust traceability from trace amounts. Proceedings of the 56th Annual Symposium on Foundations of Computer Science. 2015. pp. 650-69. https://doi.org/10.1109/FOCS.2015.46.

[28] Knoppers B (2014). International ethics harmonization and the Global Alliance for Genomics and Health. Genome Med.

[29] Torres-Espanol M, Anvar SY, Sobrido MJ (2016). Variations in the genome: the mutation detection 2015 meeting on detection, genome sequencing, and interpretation. Hum Mutat.

[30] Shringarpure SS, Bustamante CD (2015). Privacy risks from genomic data-sharing beacons. Am J Hum Genet.

[31] iDASH Privacy and Security Workshop. URL: http://www.humangenomeprivacy.org/2016/. Accessed 4 June 2017.

[32] Raisaro JL, Tramer F, Ji Z, Bu D, Zhao Y, Carey K, Lloyd D; Sofia H, Baker D, Flicek P, Shringarpure SS, Bustamante CD, Wang S, Jiang X, Ohno-Machado L, Tang H, Wang X, Hubaux JP. Addressing Beacon re-identification attacks: quantification and mitigation of privacy risks. Journal of the American Medical Informatics Association. 2017; doi:10.1093/jamia/ocw167. 10.1093/jamia/ocw167 PMC5881894 28339683

[33] Wan Z, Vorobeychik Y, Xia W, Clayton EW, Kantarcioglu M, Malin B (2017). Expanding access to large-scale genomic data while promoting privacy: a game theoretic approach. Am J Hum Genet.

